# Genomic Prediction Accuracy of Seven Breeding Selection Traits Improved by QTL Identification in Flax

**DOI:** 10.3390/ijms21051577

**Published:** 2020-02-25

**Authors:** Samuel Lan, Chunfang Zheng, Kyle Hauck, Madison McCausland, Scott D. Duguid, Helen M. Booker, Sylvie Cloutier, Frank M. You

**Affiliations:** 1 Ottawa Research and Development Centre, Agriculture and Agri-Food Canada, Ottawa, ON K1A 0C6, Canada; slanftw@gmail.com (S.L.); chunfang.zheng@canada.ca (C.Z.); kyle.hauck@canada.ca (K.H.); madison.mccausland@canada.ca (M.M.); 2 Department of Mathematics and Statistics, University of Waterloo, Waterloo, ON N2L 3G1, Canada; 3 Department of Plant Sciences, University of Manitoba, Winnipeg, MB R3T 2N2, Canada; 4 Morden Research and Development Centre, Agriculture and Agri-Food Canada, Morden, MB R6M 1Y5, Canada; scott.duguid@canada.ca; 5 Crop Development Centre, University of Saskatchewan, Saskatoon, SK S7N 5A8, Canada; helen.booker@usaska.ca

**Keywords:** flax, genome-wide association study (GWAS), single nucleotide polymorphism (SNP), genomic selection, prediction accuracy, quantitative trait loci (QTL), quantitative trait nucleotides (QTNs)

## Abstract

Molecular markers are one of the major factors affecting genomic prediction accuracy and the cost of genomic selection (GS). Previous studies have indicated that the use of quantitative trait loci (QTL) as markers in GS significantly increases prediction accuracy compared with genome-wide random single nucleotide polymorphism (SNP) markers. To optimize the selection of QTL markers in GS, a set of 260 lines from bi-parental populations with 17,277 genome-wide SNPs were used to evaluate the prediction accuracy for seed yield (YLD), days to maturity (DTM), iodine value (IOD), protein (PRO), oil (OIL), linoleic acid (LIO), and linolenic acid (LIN) contents. These seven traits were phenotyped over four years at two locations. Identification of quantitative trait nucleotides (QTNs) for the seven traits was performed using three types of statistical models for genome-wide association study: two SNP-based single-locus (SS), seven SNP-based multi-locus (SM), and one haplotype-block-based multi-locus (BM) models. The identified QTNs were then grouped into QTL based on haplotype blocks. For all seven traits, 133, 355, and 1208 unique QTL were identified by SS, SM, and BM, respectively. A total of 1420 unique QTL were obtained by SS+SM+BM, ranging from 254 (OIL, LIO) to 361 (YLD) for individual traits, whereas a total of 427 unique QTL were achieved by SS+SM, ranging from 56 (YLD) to 128 (LIO). SS models alone did not identify sufficient QTL for GS. The highest prediction accuracies were obtained using single-trait QTL identified by SS+SM+BM for OIL (0.929 ± 0.016), PRO (0.893 ± 0.023), YLD (0.892 ± 0.030), and DTM (0.730 ± 0.062), and by SS+SM for LIN (0.837 ± 0.053), LIO (0.835 ± 0.049), and IOD (0.835 ± 0.041). In terms of the number of QTL markers and prediction accuracy, SS+SM outperformed other models or combinations thereof. The use of all SNPs or QTL of all seven traits significantly reduced the prediction accuracy of traits. The results further validated that QTL outperformed high-density genome-wide random markers, and demonstrated that the combined use of single and multi-locus models can effectively identify a comprehensive set of QTL that improve prediction accuracy, but further studies on detection and removal of redundant or false-positive QTL to maximize prediction accuracy and minimize the number of QTL markers in GS are warranted.

## 1. Introduction

Genomic selection (GS) is a form of marker-assisted selection (MAS) that predicts genomic estimated breeding values (GEBVs) of test individuals through the use of genome-wide markers [1,2]. GS has been implemented in crop breeding to increase selection accuracy, reduce breeding cost, and speed-up genetic progress [3,4]. In a practical GS scheme, many factors affect its accuracy: training populations, statistical models, molecular markers, relatedness of the training populations and selection (test) populations, and so on [1,3]. Markers are one of the critical factors. In the initial concept of GS, high-density genome-wide random markers were used in genomic modeling [2]. With advances in next generation sequencing technologies and genotyping methods such as genotyping-by-sequencing (GBS) and single nucleotide polymorphism (SNP) arrays, a sufficiently large set of high-density genome-wide markers for a genetic panel can be easily generated at a low cost. However, the cost associated with obtaining such a large number of markers in the test lines can be excessive considering their generally large number. In fact, only a few markers may be associated with the traits of interest in a set of high-density genome-wide markers. This not only leads to the “large *p,* small *n*” problem [1], where a high number of marker effects need to be estimated using a population of very small sample size (*p* >> *n*), but also results in background noise in model construction because of uncorrelated markers, contrarily decreasing the genomic prediction accuracy of GS models [5]. Previous studies have confirmed that increasing marker density ensures the maintenance of association between markers and quantitative trait loci (QTL) to obtain a high prediction accuracy, but prediction accuracy plateaus when marker density increases to a certain threshold [5,6,7]. Using QTL associated with traits of interest, instead of using a full set of random SNPs in a GS model, greatly reduces the number of markers, which in turns reduces the cost of genotyping large breeding populations. Additionally, the exclusive use of markers associated with traits in GS models can increase prediction accuracy through reducing the background noise in the model construction [5,8]. Our previous study on pasmo resistance in flax has showed that using 500 QTL identified through single-locus and multi-locus genome-wide association study (GWAS) models [9] from a flax core collection (a germplasm population) [10,11] was highly effective for GS and generated a prediction accuracy as high as 0.92 compared with 0.67 when using 52,347 random SNPs [5]. 

The traditional GWAS methods, such as the general linear model (GLM) [12] and the mixed linear model (MLM) [13], are single-locus models that test the significance of marker–trait association one marker at a time and declare significant associations based on a stringent multiple-test correction (most often Bonferroni). Because of the high significance stringency, these methods only detect a few relatively large-effect quantitative trait nucleotides (QTNs) and, they lack the power to identify small-effect polygenes for more complex quantitative traits. Thus, alternative multi-locus methods have been proposed [14], including the multi-locus random-SNP-effect mixed linear model (mrMLM) [9,15], the FAST multi-locus random-SNP-effect EMMA (FASTmrEMMA) [16], the polygene-background-control-based least angle regression plus empirical Bayes (pLARmEB) [17], the iterative modified-sure independence screening EM-Bayesian LASSO (ISIS EM-BLASSO) [18], and the integration of the Kruskal–Wallis test with empirical Bayes under polygenic background control (pKWmEB). These methods adapt statistical models that simultaneously test multiple markers and, doing so, substantially increase the statistical power while simultaneously reducing Type 1 errors and running time [9,15,16,17,18,19]. These methods also usually adapt LOD scores (usually LOD ≥ 3), rather than the stringent Bonferroni correction (0.05/number of SNPs) [19], thus empowering the detection of more large and small effect QTNs [10]. In contrast to these multi-locus models, the fixed and random model circulating probability unification (FarmCPU) [20] still uses Bonferroni correction and mostly detects a few large-effect QTNs [10]. The above two types of GWAS models can be described as SNP-based single-locus (SS) and SNP-based multi-locus (SM) models. Another type of GWAS is haplotype-block-based (BM) GWAS models. Close SNPs are more likely to be inherited together; haplotype blocks are important in genetic studies [21], such as diversity studies [22], GWAS, and genomic selection [23,24,25]. The use of haplotypes in the genomic prediction of traits of allogamous plants can increase its predictive ability by 20% [23]. A restricted two-stage multi-locus multi-allele GWAS (RTM-GWAS) procedure [26] is one recently proposed BM [27,28,29]. This method first generates SNP LD blocks (SNPLDB) and then groups SNPs into an SNPLDB based on LD blocks. Each block as a marker may contain one or more SNPs that result in two or more haplotypes as its alleles for QTL mapping [26]. Thus, the significantly associated SNPLDB markers (blocks or singletons) are directly considered QTL. All these methods offer promise to identify an exhaustive set of QTNs/QTL for breeding selection. 

The objectives of this study were to evaluate GS prediction accuracies for seven major breeding selection traits using QTL identified by different GWAS models of a genetic panel of 260 flax breeding lines derived from bi-parental populations. Ten statistical GWAS models belonging to the SS, SM, and BM classes were compared to first optimize QTL identification and second to maximize prediction accuracy. 

## 2. Results

### 2.1. Phenotyping of the Population

**Figure 1:**
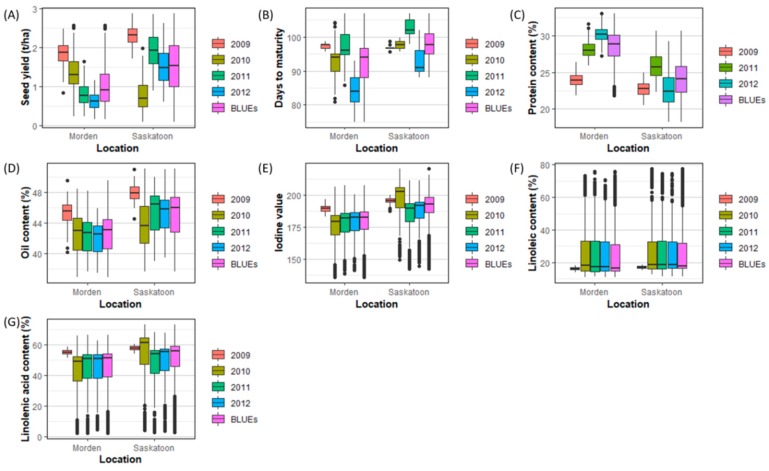
Boxplots of phenotypic data of the seven traits: seed yield (YLD) (**A**), days to maturity (DTM) (**B**), protein content (PRO) (**C**), oil content (OIL) (**D**), iodine value (IOD) (**E**), linoleic acid content (LIO) (**F**), and linolenic acid content (LIN) (**G**). BLUEs, best linear unbiased estimates across four years.

Seven breeding selection traits in flax, namely, seed yield (YLD), days to maturity (DTM), iodine value (IOD), protein content (PRO), oil content (OIL), linoleic acid content (LIO) and linolenic acid content (LIN) were measured from 260 lines from bi-parental populations grown in the field for four years at two locations (Figure 1). Less variability was observed in 2009 at both locations across all traits because only 96 of the 260 lines were evaluated that year at the two locations. DTM, PRO, and YLD showed significant differences across four years and both locations, whereas the seed quality traits (IOD, LIN, LIO, and OIL) had relatively similar performance at the two locations. All traits, with the exception of PRO, had significantly higher values in Saskatoon than Morden (*p* < 2 × 10^−16^ for all six traits except for PRO). The analysis of variance also showed a significant interaction between years and locations for all traits except for LIO (*p* = 0.97; Table S1). The performance of the seven traits in different years and locations suggested that the phenotypic data of each environment (years and locations) should be used to identify all potential stable and environment-specific QTNs associated with the traits. 

### 2.2. Haplotype Blocks

RTM-GWAS was used to identify haplotype blocks of 17,277 SNPs in the 260 lines [26]. A total of 2776 haplotype blocks with two or more SNPs per block and 2852 singletons were generated. Although a singleton has only one SNP, it can be treated as an independent block. As such, a total of 5628 haplotype blocks were considered for further QTL mapping and analyses. The number of blocks ranged from 231 in chromosome 11 (Lu11) to 500 in chromosome 1 (Lu1) with an average block size of 20.09–29.78 kb (Table 1). 

### 2.3. QTNs/QTL 

To compare the performance of different statistical models to identify QTNs in GWAS, three types of models were evaluated: (1) two SS models, including GLM [12] and MLM [13], (2) seven SM models, including the six models implemented in the mrMLM package and FarmCPU implemented in the MVP package, and (3) the BM model, RTM-GWAS [26]. 

**Figure 2:**
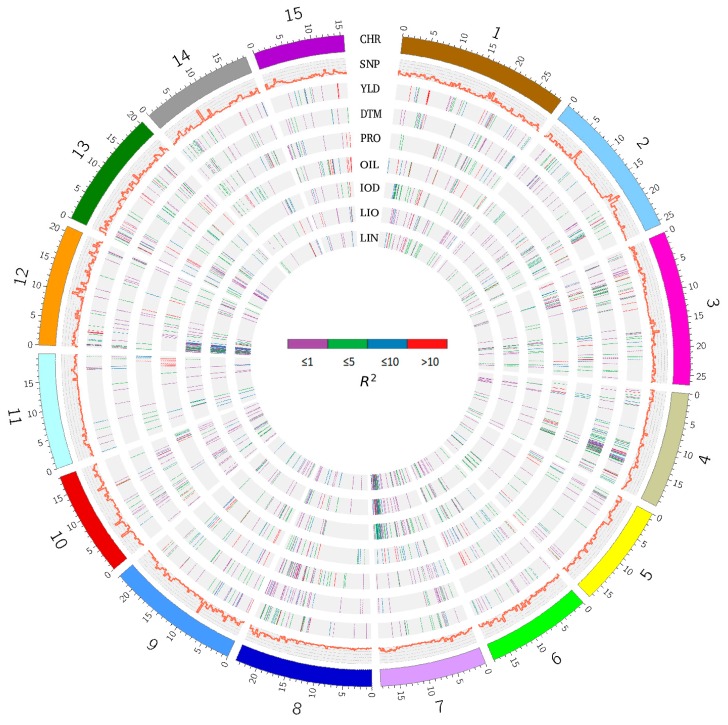
Circos map of quantitative trait nucleotides (QTNs) associated with seven traits in the 260 lines. Track 1 (from outer), chromosomes; Track 2, density of 17,277 SNPs (bin size of 300 kb); Track 3, QTNs for YLD; Track 4, QTNs for DTM; Track 5, QTNs for PRO; Track 6, QTNs for OIL; Track 7, QTNs for IOD; Track 8, QTNs for LIO; Track 9, QTNs for LIN. The effects of QTNs are represented by different colors. *R^2^* ≤ 1%, purple; 1% < *R^2^* ≤ 5%, green; 5% < *R*^2^ ≤ 10%, blue; *R^2^* > 10%, red. YLD, seed yield; DTM, days to maturity; PRO, protein content; OIL, oil content; IOD, iodine value; LIO, linoleic acid content; LIN, linolenic acid content; SNP, single nucleotide polymorphism.

A total of 268 and 407 unique QTNs for the seven traits were identified using SS and SM, totaling 608 unique QTNs, while 1208 significant haplotype blocks or singletons were detected using BM (RTM-GWAS) (Table 2, Tables S2 and S3). The QTNs from SS and SM were further grouped based on haplotype blocks; that is, the QTNs located in the same haplotype block were grouped into a QTN cluster or a QTL. As such, 608 QTNs for the seven traits identified using SS and SM were grouped into 427 unique QTN clusters or QTL for the seven traits. Since the results from RTM-GWAS were haplotype-block-based, they were directly treated as QTL. Therefore, 1420 unique QTL were identified for the seven traits when all models (SS+SM+BM) were considered, including 361, 351, 269, 254, 283, 254, and 256 QTL for YLD, DTM, PRO, OIL, LOD, LIO, and LIN, respectively (Table 2, Figure 2). For each QTL, a tag QTN was selected to represent the QTL.

**Figure 3:**
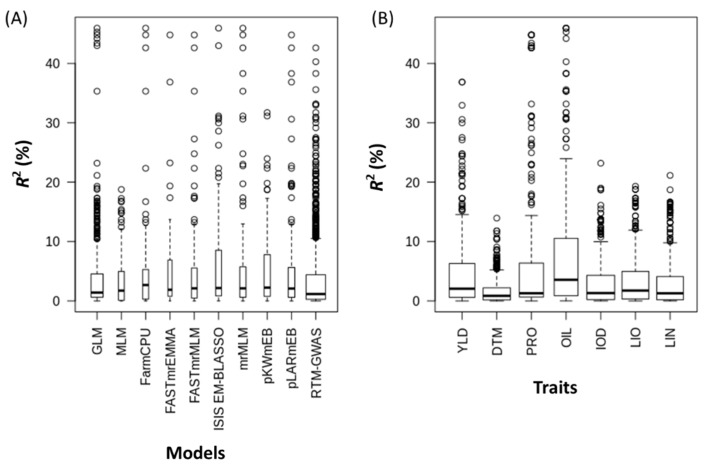
Boxplots of allele effects (*R*^2^) of quntitativ trait loci (QTL) for ten genome-wide association study (GWAS) models (**A**) and seven phenotypic traits (**B**). YLD, seed yield; DTM, days to maturity; PRO, protein content; OIL, oil content; IOD, iodine value; LIO, linoleic acid content; LIN, linolenic acid content.

The allelic effects of all QTL are illustrated and summarized in Figure 2 and Figure 3, and Table 2, Tables S2 and S3. Similar QTL effects were observed among the ten statistical models (Figure 3A, Table S3). Using *R^2^* ≥ 5% as the criterion to define major QTL, 520 of the 1420 unique QTL would be considered major, explaining 12.06 ± 8.24% of the variance. QTL for PRO, OIL, and YLD had relatively larger effects than those of the other four traits (Figure 3B and Table 2). The number of QTL for YLD and OIL exceeded that of the other traits, being 110 (30.5%) and 111 (43.7%), respectively, while the smallest number of major QTL belonged to DTM with 36 out of 351 (10.3%). 

**Figure 4:**
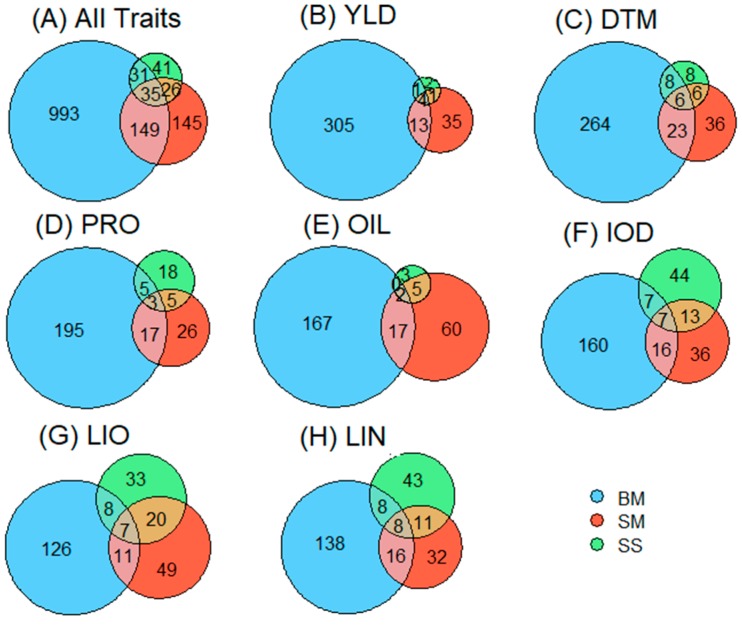
Venn diagrams of quantitative trait loci (QTL) identified by three types of genome-wide association study (GWAS) models for all seven traits (**A**) and individual traits (**B**–**H**). SS, SNP-based single-locus models; SM, SNP-based multi-locus models; BM, haplotype-block-based multi-locus model. YLD, seed yield; DTM, days to maturity; PRO, protein content; OIL, oil content; IOD, iodine value; LIO, linoleic acid content; LIN, linolenic acid content; SNP, single nucleotide polimorphsm.

The GWAS models identified different sets of QTL (Figure 4, Tables S2 and S4). BM detected four times more QTL than the SS+SM and most differed from one another. Of the 1420 QTL, only 215 QTL were shared by both SS+SM and BM, ranging from 18 out of 361 QTL for YLD (5%) to 32 out of 256 QTL for LIN (12.5%). The average allele effect (*R*^2^) of the shared QTL among the three types of models was 2.75%, whereas QTL that were not shared had *R*^2^ of 2.73% for BM, 3.16% for SM, and 2.62% for SS, showing that the shared QTL did not necessarily have greater QTL effects. Between the SNP-based models (SS and SM), the six SM models had more QTL in common with BM than the two SS models (GLM and MLM). SS identified fewer QTL for YLD, DTM, PRO, OIL, and LIO than SM, but a similar number was identified by the two model types for IOD and LIN. 

**Figure 5:**
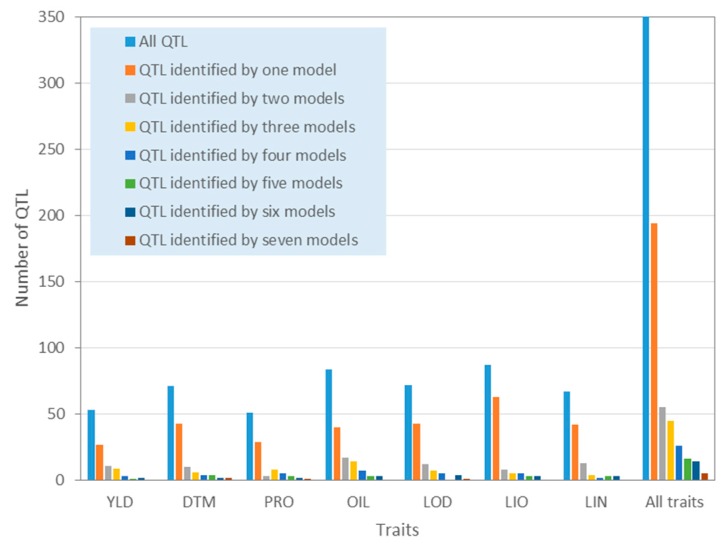
Histograms of quantitative trait loci (QTL) that were identified by one of the seven SNP-based multi-locus models or simultaneously by two or more models for the seven traits. YLD, seed yield; DTM, days to maturity; PRO, protein content; OIL, oil content; IOD, iodine value; LIO, linoleic acid content; LIN, linolenic acid content; SNP, single nucleotide polimorphsm.

Similarly, seven SNP-based multi-locus models also identified different sets of QTL (Figure 5, Tables S3 and S4). For all seven traits, a total of 355 unique QTL were obtained using the seven SM models (Table 2). Models pKWmEB, pLARmEB and pLARmEB identified 133, 130, and 121 QTL, respectively, followed by ISIS EM-BLASSO (133), FASTmrMLM (96), and FarmCPU (96). FASTmrEMMA identified the fewest QTL (52). More than half of the QTL (an average 58% across the seven traits) identified by the seven SM models were detected by different single models, varying from different traits, ranging from 47.6% (OIL) to 72.4% (LIO). The remaining 42% of the QTL were simultaneously identified by two or more models. Out of 355 QTL, 194 (54.7%), 55 (15.5%), 45 (12.7%), 26 (7.3%), 16 (4.5%), 14 (3.9%), and 5 (1.4%) were identified by a single, two, three, four, five, six, and seven models, respectively. These results indicated that the seven SM models are complementary in QTL identification. 

### 2.4. Pleiotropic QTL

**Figure 6:**
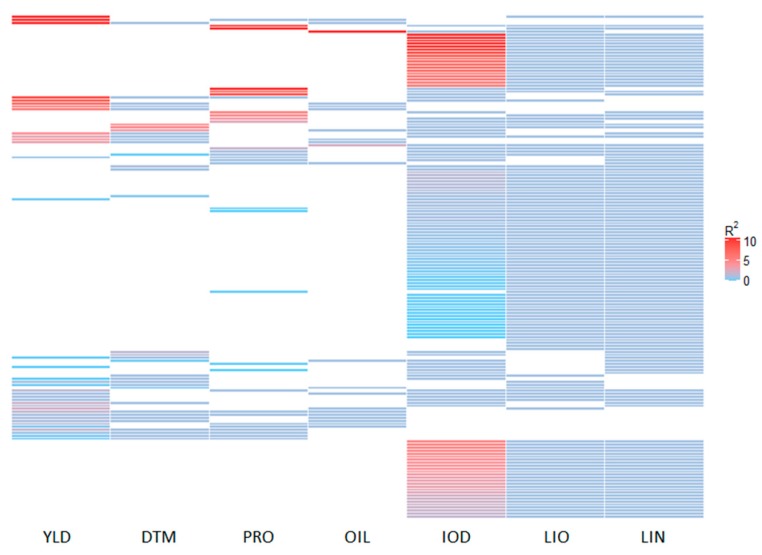
Heatmap of pleiotropic effects of 168 quantitative trait loci (QTL) associated with three or more traits. YLD, seed yield; DTM, days to maturity; PRO, protein content; OIL, oil content; IOD, iodine value; LIO, linoleic acid content; LIN, linolenic acid content.

Of the 1420 unique QTL identified with all models, 407 were pleiotropic with effects on two or more traits, of which, 239, 139, 25, and 4 QTL were simultaneously associated with 2, 3, 4, and 5 traits, respectively. Some QTL for YLD were associated with DTM as well as PRO and OIL, while many QTL for IOD, LIO, and LIN were co-located (Figure 6). Table 3 lists the number of QTL shared between any two traits. More than 50% of the QTL were shared between any two of LIO, LIN, and IOD. YLD and DTM also had 19% of their respective QTL in common. 

### 2.5. Genomic Prediction Accuracy 

To define the marker sets that generate the best prediction accuracy, we constructed GS models for the seven traits using GBLUP with three types of markers (all SNPs, QTL of all the traits, and QTL of single traits). The QTL marker sets were obtained from four different combinations of GWAS models (SS, SS+SM, BM, and all models, i.e., SS+SM+BM). For the marker type “All SNPs” or the “QTL of all traits”, the same 17,277 SNPs or the same set of QTL of all seven traits (133, 427, 1208, and 1420 QTL for SS, SS+SM, BM, and SS+SM+BM, respectively; Table 2) were used for GS model construction of each trait. However, for the marker type “QTL of single traits”, the specific QTL sets for the respective traits were used as marker sets (Table 2). A joint analysis of variance (ANOVA) of prediction accuracy (*r*) for three factors, namely, traits, GWAS models, and types of markers, was performed. The ANOVA results showed significant differences among traits, marker types, or marker sets due to GWAS models, as well as interactions between the three factors (Table S5). 

**Figure 7:**
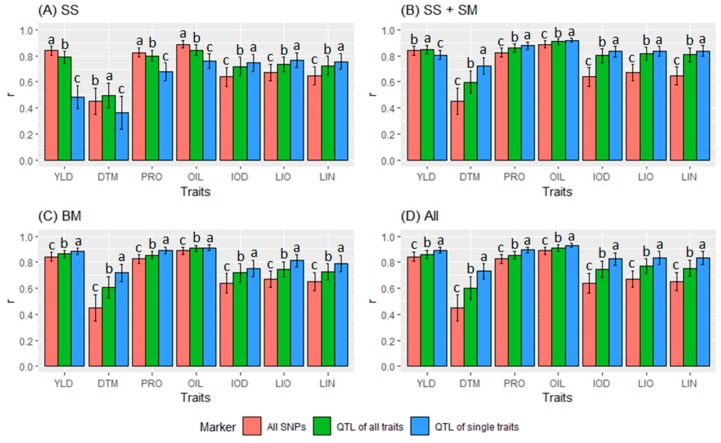
Comparisons of genomic prediction accuracy (*r* ± *s*) using different marker sets, including all single nucleotide polymorphisms (SNPs) and quantitative trait locus (QTL) sets identified by different statistical models: (**A**) SNP based single-locus model (SS), (**B**) SS + SNP based multi-locus model (SM), (**C**) haplotype-block-based model (BM), and (**D**) all three models of SS+SM+BM (All). For each trait, three marker sets were compared for prediction accuracy: All SNPs, QTL of all traits (QTL together for all seven traits), and QTL of single traits (QTL for individual traits). Different letters represent statistical significance of *r* values among different types of markers within each trait. A tag quantitative trait nucleotide (QTN) for each QTL was used for analyses. YLD, seed yield; DTM, days to maturity; PRO, protein content; OIL, oil content; IOD, iodine value; LIO, linoleic acid content (LIO); LIN, linolenic acid content.

**Figure 8:**
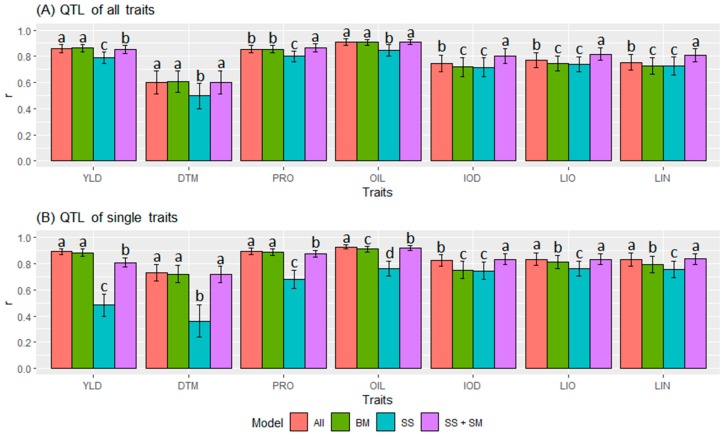
Comparisons of genomic prediction accuracy (*r* ± *s*) by different statistical models, including SNP-based single-locus model (SS), SS+SNP-based multi-locus model (SM), haplotype-block-based model (BM), and all three models of SS+SM+BM (All), which were used for quantitative trait locus (QTL) identification. (**A**) QTL of all traits were used for GS, and (**B**) QTL of single traits were used for GS. A tag quantitative trait nucleotide (QTN) for each QTL was used for analyses. For each trait, different letters represent statistical significance of *r* values among different GWAS models. YLD, seed yield; DTM, days to maturity; PRO, protein content; OIL, oil content; IOD, iodine value; LIO, linoleic acid content (LIO); LIN, linolenic acid content; SNP, single nucleotide polymorphism.

Among the seven traits, the GS models generated the highest *r* for OIL (0.887 ± 0.058), following by PRO (0.838 ± 0.072), YLD (0.808 ± 0.126), LIO (0.776 ± 0.074), LIN (0.765 ± 0.083), IOD (0.753 ± 0.085), and DTM (0.588 ± 0.150). They were all significantly different from each other at a 0.05 probability level. This trend was consistently observed in terms of QTL identified by different GWAS models (Figure 7) and in terms of QTL of all or single traits (Figure 8).

Among the three types of markers, the GS models with the QTL markers (either QTL of all traits or QTL of single traits) identified by SS+SM, BM or all models had significantly greater *r* values than those with all SNPs for all seven traits (Figure 7B–D). An exception was for YLD, DTM, PRO, and OIL when QTL identified by SS were used (Figure 7A). The GS models using single-trait QTL identified by SS+SM (Figure 7B), BM (Figure 7C) or all models (SS+SM+BM) (Figure 7D) performed significantly better than those using QTL of all traits. The average *r* values of the seven traits were 0.789 ± 0.155, 0.774 ± 0.116, and 0.709 ± 0.134 when using QTL of single traits, QTL for all traits, and all SNPs, respectively, and they all significantly differed from each other.

Since more pleiotropic QTL were found between YLD and DTM, between PRO and OIL, and among IOD, LIO, and LIN, we also compared prediction accuracy for all SNPs, single-trait QTL, and the combined QTL of YLD+DTM, PRO+OIL, and IOD+LIO+LIN identified by all statistical models (Table 4). The results showed that the combined marker sets of two or three traits yielded a slightly higher *r* estimates for LIO only, but similar or slightly lower estimates than the ones obtained using the single-trait QTL markers. This indicated that using QTL from more traits did not improve prediction accuracy. Using single-trait QTL marker sets in GS yielded significantly better prediction accuracy.

In terms of QTL marker sets generated by different GWAS models, SS did not identify sufficient QTL markers from YLD, DTM, PRO, and OIL, thus, resulting in low *r* values for these four traits (Table 4, Figure 7A). All GS models using QTL by SS generated lower *r* values than those using QTL by BM, SS+SM, or all models for all seven traits (Table 4, Figure 8) except IOD, LIO, and LIN with all-trait QTL (Figure 8A) and IOD with single-trait QTL (Figure 8B). 

BM and SS+SM are two different types of GWAS models. The GS models with QTL identified by SS+SM outperformed BM for IOD, LIN, LIO, and OIL or had similar prediction accuracy for DTM with BM. However, for YLD, BM consistently outperformed SS+SM. For PRO, SS+SM had similar or better performance when all-trait QTL were used (Figure 8A). For the most part, the all-model (SS+SM+BM) had similar to or better results than SS+SM or BM independently (Figure 8, Table 4). Due to significant interactions between marker types and marker sets (Table S5), the GS models with the best prediction accuracy were those using QTL of single traits identified by all GWAS models (SS+SM+BM) for OIL (0.929 ± 0.016), PRO (0.893 ± 0.023), YLD (0.892 ± 0.030), and DTM (0.730 ± 0.062), and by SS+SM for LIN (0.837 ± 0.053), LIO (0.835 ± 0.049), and IOD (0.835 ± 0.041).

In this study, the seven traits were phenotyped in two locations, Morden and Saskatoon, which are representative of the production areas of oilseed flax in Western Canada. To assess the effect of location on genomic prediction and whether or not separate GS models should be constructed in terms of different locations, we compared the prediction accuracy of models using the phenotypic values obtained in Morden and Saskatoon as well as the BLUEs calculated over both locations for the three different types of markers and the seven traits. Only the GS models for YLD at Saskatoon and PRO at Morden performed significantly better than the others. For all other traits, the prediction accuracies were similar regardless of the location-based data set (Table 5 and Table S6). Single-trait QTL for all seven traits as markers significantly improved prediction accuracy compared to all SNPs or all-trait QTL in terms of different locations (Table 5). For all seven traits, the GS models with single-trait QTL had significantly greater prediction accuracy than those with all SNPs or all-trait QTL (Table 5).

## 3. Discussion

A good training population in GS has a strong relationship with the test populations in breeding and may include germplasm genotypes for parent selection or breeding lines for offspring selection. In the present study, all lines used for GS evaluation were derived from three bi-parental crosses [30,31]. The two parents of the first cross were Canadian high-yielding conventional linseed cultivars with high LIN of 55–57% (CDC Bethune and Macbeth). The second population resulted from a cross between a low LIN breeding line (E1747) and a European fiber flax cultivar with ~55% LIN (Viking). The third cross had two parents of a yellow-seeded and low LIN (2–3%) cultivar (Solin^TM^ SP2047) and a high LIN breeding line with 63–66% LIN (UGG5-5). Therefore, this genetic panel exhibited diversity in genetic variation in major breeding selection traits [30,31]. Although these breeding lines were derived from a few parents, they are close to breeding populations. Therefore the results obtained herein apply to practical breeding. 

Given a training population in practical breeding, markers will be a critical factor for improving prediction accuracy since GS predicts breeding values of selection traits using a set of markers [2]. Prediction accuracy directly assesses the efficiency of a marker set in GS. Here, using prediction accuracy, we consistently demonstrated that QTL markers outperformed genome-wide random SNPs for GS of any traits, further confirming and validating the results observed for pasmo resistance using a flax core germplasm collection of 370 accessions [5]. The use of QTL identified by GWAS models significantly increased prediction accuracy for all seven traits, from 4% for OIL (from 0.89 to 0.93) to 29% for DTM (from 0.45 to 0.73) compared to genome-wide random SNPs (Table 4). The reasons that QTL outperformed genome-wide random SNPs are likely a reduction in background noises or as a consequence of reduced multi-collinearity due to the removal of unrelated markers. 

Many statistical models of GWAS have been proposed to identify QTL. In this study, we investigated three types of models, including two SS, seven SM, and one BM, totaling ten different models. However, it seemed that different models generated varying sets of QTL in which only a small portion of QTL was shared by two or more models (Figure 4 and Figure 5, Table S4). Similar results were also obtained in the previous study of QTL identification for pasmo resistance in flax, where the same SS and SM models were used [10]. The two SS methods (GLM and MLM) identified only 133 QTL for all seven traits, accounting for 9% of 1420 QTL, whereas the seven SM methods identified 355 QTL, accounting for 25% of the total QTL. One haplotype block-based model, RTM-GWAS, identified a total of 1208 QTL alone (85%), three times the total QTL identified by the nine SNP-based models (SS+SM). A haplotype-block-based GWAS is expected to increase power relative to SNP-based approaches, resulting in a higher number of QTL identified. First, the block-based approach reduces the dimension of association testing when a single global test for a block is used and thus preserves power and helps maintain reasonable false-positive rates. Second, a haplotype method also captures associations of nearby SNPs that would have been otherwise missed with an SNP-by-SNP approach [32]. Because different algorithms and assumptions are adopted in different models, their QTL results may be complementary in GS. 

We evaluated the performance of different sets of QTL markers identified by different models via prediction accuracy. The results indicated that two SS models did not identify sufficient QTL for YLD, DTM, PRO, and OIL, resulting in low prediction accuracy as compared with all SNPs, whereas SS+SM+BM or SS+SM identified sufficient QTL to yield the highest prediction accuracies for all seven traits, strongly suggesting that the advantages of different statistical models are complementary and the combined results from different models improve prediction accuracy. In terms of the number of QTL identified and prediction accuracy, the combined use of SNP-based models (SS+SM) was superior to other models or their combinations since only a small number of QTL were identified by SS+SM compared to BM, but similar or better prediction accuracies were obtained for most traits. The QTL identified by BM was three times greater than those identified by SS+SM, but BM significantly outperformed SS+SM only for YLD and PRO. While BM and SS+SM had similar prediction accuracies for DTM, SS+SM was significantly superior to BM for the remaining four traits: OIL, IOD, LIO, and LIN (Table 4). These results implied that the combined use of different GWAS models facilitates the identification of a potentially complete set of QTL associated with the traits, but some of them may be redundant or possibly false positives. Therefore, further investigations to design a methodology to identify and remove the redundant or false-positive QTL that would maximize prediction accuracy and minimize the number of QTL markers in GS are warranted. 

The heritability of a trait is an important factor that affects the efficiency of genomic selection over traditional phenotypic selection. Generally, high prediction accuracies are more easily achieved with high heritability traits [8]. Conversely, genomic selection is likely more beneficial for traits with low heritability [33,34]. In this study, the broad-sense heritability ($H^{2}$), representing the extent with which the performance of a trait is affected by the environment, was estimated for the seven traits (Table 6). Compared to the maximum prediction accuracy (*r*) of each trait, the $H^{2}$ of the traits did not exhibit a consistent relationship with prediction accuracy. OIL with a moderate estimate (0.69) produced the highest prediction accuracy (0.93). The three fatty acid composition related traits, LIO, LIN, and IOD, had a relatively high $H^{2}$ values (0.81–0.83) and a similarly high prediction accuracy. Albeit with low heritability, YLD (0.44) and PRO (0.20) generated the second-highest prediction accuracy (0.89). However, considering the relative efficiency of genomic prediction over phenotypic selection (*RE*), which is defined as *r*/$\;H^{2}$ [35], the traits with a low $H^{2}$ had a high *RE*, exhibiting a strong negative correlation (Table 6). Especially YLD with $H^{2}$ of 0.2 generated as high as 4.45 times selection efficiency over phenotypic selection, demonstrating more benefits of GS for low heritability traits. Based on *RE*, GS for YLD, DTM, PRO and OIL outperformed phenotypic selection, whereas GS for IOD, LIO and LIN were equivalent to or slightly better than phenotypic selection. A similar trend for YLD, OIL, IOD, LIO and LIN was also observed when a limited number of microsatellite markers were used [30]. Compared to $H^{2}$, the prediction accuracy of a trait was more dependent on genomic heritability that represents a proportion of additive genetic variation explained by the markers (Table 6). In other words, prediction accuracy mostly depends on whether the marker set contains sufficient QTL to contribute to the total variation of the phenotypes, or whether all related QTL have been identified from the marker set if QTL markers are used in GS models. 

Pleiotropy of genes has been thought to be the molecular basis of trait genetic correlation. We have identified highly significant correlations between YLD and DTM, between PRO and OIL, and among IOD, LIO, and LIN (Table S7) [30,31]. Correspondingly, we also identified many pleiotropic QTL between these traits in the present (Table S2 and Table 3, Figure 5) and previous studies [31], suggesting that different traits may be genetically controlled by the same or tightly linked genes/QTL. Our hypothesis is that if some QTL are pleiotropic to two or more traits, all the QTL identified from genetically-related traits could be used as markers in GS to improve prediction accuracy. Therefore, we evaluated GS accuracy of different marker sets, including QTL of single traits, QTL of all seven traits, and QTL of some combinations of related traits (YLD+DTM, PRO+OIL, IOD+LIO+LIN). Our results rejected the hypothesis, indicating that QTL from pleiotropic traits did not improve GS accuracy for any of the seven traits. However, this does not necessarily signify that the pleiotropic QTL do not have a role in improving GS accuracy because QTL identified from each single trait already includes QTL pleiotropic to other traits and additional unrelated QTL from other traits thereby reducing prediction accuracy as a consequence of redundancy or background noise. Thus, our results strongly suggest that QTL from single traits can not only significantly improve prediction accuracy but also reduce the number of markers, which in turn would decrease genotyping cost in practical breeding programs compared with the use of all SNPs or QTL of all traits or QTL of any trait combinations. 

Significant genotype by environment (GXE) interactions (Table S1, Figure 1) hinted at the potential need for separate GS models for different breeding target regions in order to maximize GS accuracy. We constructed separate GS models for two locations: Saskatoon and Morden, using phenotypic data observed from the two locations as well as GS models using BLUEs over years and locations. Only the GS models for YLD at Saskatoon and PRO at Morden had higher GS accuracies than any of the other models because these two traits had the largest GXE interaction, although significant GXE interactions also existed for the other five traits (Table S1). This suggested that genomic selection based on BLUEs over years and locations is suitable for traits with moderate or no GXE, but higher accuracies are obtained if GS is performed using by location for traits with high GXE. 

GS applied in practical breeding requires not only a high prediction accuracy but also an acceptable cost. Although GBS is a most popular genotyping approach to obtain high density genome-wide random SNPs, it is not an efficient genotyping approach for GS. It generates a large number of unused SNPs. The cost is also a limiting factor for a GS scheme with a large genome, such as wheat. In addition, it is prone to generate missing data in low-coverage sequencing. Recently, some new target-oriented genotyping methods have been developed for breeding, such as genotyping by target sequencing (GBTS) [36], and RAD capture (Rapture) [37]. These methods enable low-cost, high-read coverage genotyping of target loci, and also allow previous training data based on non-captured GBS to be fully compatible with new rapture data [38]. Using GBTS, for example, only USD 12.36 per sample for 5000 target markers of the 2.3 Gb maize genome was needed [36], a much cheaper option than GBS [4,39]. The Rapture assay consistently outperformed the GBS assay, and its cost per sample was approximately 40% less than GBS in oat, a crop with a genome size of 12.5 GB [38]. Therefore, QTL identification by single-locus and multi-locus GWAS models combined with new target-oriented genotyping methods facilitate the implementation of a highly efficient genomic selection scheme in modern plant molecular breeding.

## 4. Materials and Methods

### 4.1. Plant Materials, SNPs and Phenotypic Data 

A total of 260 lines derived from three different bi-parental populations was used as a genotype panel for the association study and genomic selection evaluation. These lines consisted of 97 F6-derived recombinant inbred lines (RILs) generated by single seed descent from a cross between two Canadian high-yielding conventional linseed cultivars CDC Bethune and Macbeth, 91 F6-derived RILs from a cross between a low LIN breeding line E1747 and a French fiber flax cultivar Viking, and 72 F1-derived doubled haploid (DH) lines obtained from a cross between two breeding lines SP2047 (low LIN, 2–3%) and UGG5-5 (high LIN, 63–66%). The details have been previously described [30,31].

Reduced representation libraries from the 260 lines were re-sequenced by the Michael Smith Genome Sciences Centre of the BC Cancer Agency, Genome British Columbia (Vancouver, BC, Canada) using 100-bp paired-end reads on an Illumina HiSeq 2000 platform (Illumina Inc., San Diego, CA, USA) as previously described [40]. The short reads were aligned to the flax scaffold sequences of cultivar CDC Bethune [41], and SNPs were called and filtered using the revised AGSNP pipeline [40,42,43]. Final SNPs with a MAF ≥ 0.01 and a genotyping rate ≥ 60% were used for further imputation using Beagle v.4.2 [44] to estimate missing data. The coordinates of all SNPs based on scaffolds were converted to the new chromosome-based flax pseudomolecules v2.0 [45]. 

All lines were evaluated in field trials over four years (2009–2012) at two sites, Morden Research and Development Centre, Manitoba (MD) and Kernen Crop Research Farm near Saskatoon, Saskatchewan (SAS) in Canada. A type-2 modified augmented design (MAD) [46] was used for the field experiments from which phenotypic data were collected. The detailed experimental design was previously described [30,31,47]. Seven major breeding selection traits were evaluated, including YLD, DTM, PRO, OIL, IOD, LIO, and LIN. The methods and criteria used for the evaluation of these traits are detailed in [31]. All phenotypic data from the field experiments and laboratory measurements were adjusted for soil heterogeneity, as previously described, based on the MAD pipeline [47]. The BLUE values over multiple environmental phenotypes estimated using TASSEL [48] were used for further association study analyses. The Shapiro–Wilk normality test was performed for all traits using the R function “shapiro.test”. All seven traits followed approximately a normal or mixed normal distribution.

### 4.2. Identification of Haplotype Blocks 

The software RTM-GWAS [26] was used in identifying haplotype blocks. RTM-GWAS provides a function module to group sequential SNPs into linkage disequilibrium blocks (SNPBDBs), using the block-partitioning approach with confidence interval based on genome-wide *D*’ pattern [49]. The software requires SNP data in VCF format. The default values for all the other parameters were used, including the minimum minor haplotype frequency (0.01), and the maximum length of blocks (100 kb).

### 4.3. QTL Identification

Three types of GWAS models were used to identify putative QTNs associated with the seven traits. These models included two traditional SNP-based single-locus models (GLM [12] and MLM [13]), seven SNP-based multi-locus models (pLARmEB, pKWmEB, FASTmrMLM, FASTmrEMMA, ISIS EM-BLASSO, and mrMLM implemented in the R package mrMLM, https://cran.r-project.org/web/packages/mrMLM/index.html, and FarmCPU [20] implemented in the R package MVP, https://github.com/XiaoleiLiuBio/MVP), and one haplotype block-based model RTM-GWAS [26]. Kinship genetic relationship matrix was estimated using the protocol suggested by each GWAS software package. The population structure of the 260 lines was estimated using principal component analysis (PCA) using TASSEL [48], and the first five principal components (PCs) accounting for 72.35% of the total variation were chosen as covariates in all GWAS models. GWAS were conducted separately for each phenotype data sets from the four individual years and two locations and the BLUE dataset over years and locations for each trait to identify all stable or environment-specific QTL. Thus, all QTNs from different phenotype data sets were merged for analyses. 

For GLM, MLM, and FarmCPU, the threshold of significant marker-trait associations was determined by a critical *p*-value (α = 0.05) subjected to Bonferroni correction, i.e., the corrected *p*-value = 2.89 × 10^−6^ (0.05/17,277 SNPs). For the six models implemented in the mrMLM R package, a log of odds (LOD) score of three was used to detect robust marker–trait association signals for these six methods. 

The identified QTNs were further grouped into QTN clusters or QTL based on the haplotype blocks generated by RTM-GWAS. The SNPs within the same block were treated as a QTN cluster or a QTL. The QTN with the largest *R*^2^ within a QTN cluster was selected as a tag QTN for that cluster or QTL. 

### 4.4. Genomic Selection (GS) Models and Evaluation

The statistical model Genomic BLUP (GBLUP) implemented in the R package BGLR [50] was used to evaluate prediction accuracy for different marker sets. The computation procedures of GBLUP have been described in detail [51,52]. When preparing QTL marker data for model construction, the positive-effect allele of the tag QTN/SNP of a QTL was coded “1” and the alternative allele “−1”. Similarly for the SNP marker set, the reference allele of an SNP was coded “1” and the alternative allele “−1”. Missing data were coded “0”. The EM algorithm implemented in the R package rrBLUP [53] was used to impute the missing marker data.

The five-fold random cross-validation was used to evaluate GS models. The 260 lines were randomly partitioned into five subsets. For a given partition, each subset was, in turn, used as test data, while the remaining four subsets were used as a training dataset. This partitioning was repeated 50 times. The accuracy of the genomic predictions (*r*) was defined by the Pearson correlation coefficient between the GBEV values predicted by GS and the observed phenotypic values. To compare GS models constructed from different markers, a joint analysis of variance with Tukey’s multiple pairwise-comparisons (HSD.test function) was performed to test the statistical significance of differences in *r* values using the R package agricolae (https://cran.r-project.org/web/packages/agricolae/index.html). 

### 4.5. Estimation of Broad-sense and Genomic Heritability

Broad-sense heritability of phenotypes for the traits was estimated using the inter-environment correlation method [54]. Genomic heritability of the traits is a molecular marker based heritability parameter that explains a portion of the additive genetic variance ($\sigma_{A}^{2}$): $h^{2}$ = $\sigma_{A}^{2}$/($\sigma_{A}^{2}$+$\sigma_{e}^{2}$). It was estimated using the R package sommer with the GBLUP model [55]. 

## 5. Conclusions

In this study, we adopted a set of genomic and phenotypic data, including 260 lines derived from bi-parental populations, 17,277 genome-wide random SNPs, and phenotypes of seven major breeding selection traits in flax, which were evaluated in four years and two locations, to find optimal markers for maximizing prediction accuracy and minimizing cost of genotyping in breeding selection for these important traits. Our results confirmed and validated that the use of QTL significantly increases prediction accuracy compared to genome-wide random SNPs and cuts down the cost of genotyping of test populations since the number of markers used in GS models have been dramatically reduced to a magnitude of dozens to hundreds rather than a scale of thousands, even hundreds of thousands. In the evaluation of GS models, we compared QTL identified by different types of GWAS models and also QTL from a single trait or QTL from all traits. The results indicated that the highest prediction accuracy of individual traits was obtained by using QTL of respective traits identified by SS+SM+BM or SS+SM, rather than using all genome-wide random markers or QTL of all seven traits. In terms of the number of QTL identified and prediction accuracy, SS+SM outperformed other models or their combinations for most traits. Our work demonstrates that the combined use of single- and multi-locus GWAS models can identify sufficient QTL of traits and significantly improve prediction accuracy, but some redundancy or false-positives may exist in QTL identified by some GWAS models, especially in those by BM. Therefore, further investigation of detection and removal of the redundant or false-positive QTL to maximize prediction accuracy and minimize the number of QTL markers in GS is warranted. 

## Figures and Tables

**Table 1:** The haplotype blocks identified from 17,277 single nucleotide polimorphsims (SNPs) in the 260 lines and association with quantitative trait loci (QTL) of traits.

Chr	No of Blocks (Including Singletons)	No of Singletons	Average SNPs Per Block	Average Block Size (Kb)	No of Blocks with QTL
Lu1	500	257	3.02	27.61 ± 32.99	126
Lu2	374	178	4.10	28.07 ± 34.68	101
Lu3	472	242	2.81	23.96 ± 30.24	116
Lu4	337	182	2.45	23.31 ± 32.50	108
Lu5	308	133	3.48	29.78 ± 35.16	57
Lu6	419	227	2.80	26.11 ± 32.91	80
Lu7	296	157	2.86	29.15 ± 35.21	116
Lu8	433	244	2.52	20.05 ± 27.18	126
Lu9	443	208	3.19	24.89 ± 31.83	95
Lu10	389	210	2.89	25.79 ± 31.75	80
Lu11	231	127	2.60	26.50 ± 33.37	44
Lu12	355	149	3.90	26.70 ± 32.72	112
Lu13	448	216	3.51	29.50 ± 34.34	111
Lu14	381	208	2.82	23.04 ± 31.60	89
Lu15	242	114	3.07	27.81 ± 33.42	59
Total	5628	2852	3.07	26.12 ± 32.64	1420

**Table 2:** Quantitative trait nucleotides (QTNs)/quantitative trait loci (QTL) identified from 17,277 single nucleotide polymorphisms (SNPs) in the 260 lines for the seven traits using three types of genome-wide association study (GWAS) models.

Trait	QTNs		QTL								
	SS	SM	SS	SM	SS+SM	BM	All (SS+SM+BM)	Major QTL	Major QTL Effect (*R*^2^, %)	Minor QTL Effect (*R*^2^, %)	All QTL Effect (*R*^2^, %)
YLD	13	58	8	53	56	323	361	110	11.03 ± 6.75	1.32 ± 1.24	4.64 ± 6.14
DTM	43	76	28	71	87	301	351	39	6.99 ± 2.11	1.12 ± 1.25	1.70 ± 2.22
PRO	66	56	31	51	74	220	269	77	16.55 ± 12.50	1.24 ± 1.25	5.48 ± 9.54
OIL	17	88	10	84	87	186	254	111	15.80 ± 10.26	1.43 ± 1.30	7.88 ± 9.96
IOD	153	82	71	72	123	190	283	55	9.47 ± 3.79	1.30 ± 1.40	2.96 ± 3.91
LIO	146	102	68	87	128	152	254	70	9.86 ± 3.98	1.40 ± 144	3.50 ± 4.34
LIN	189	127	70	67	118	170	256	53	10.21 ± 4.10	1.25 ± 1.37	3.06 ± 4.22
All	268	407	133	355	427	1,208	1,420	520	12.06 ± 8.24	1.28 ± 1.33	3.99 ± 6.34

SS, SNP-based single-locus models; SM, SNP-based multi-locus models; BM, haplotype-block-based multi-locus model. Major QTL are defined as *R*^2^ ≥ 5%, while minor QTL as *R*^2^ < 5%.

**Table 3:** Numbers of quantitative trait loci (QTL) that were pleiotropic on any two of the seven traits.

Trait	YLD	DTM	PRO	OIL	IOD	LIO	LIN
YLD	361	69(19.1,19.7)	28(7.8,10.4)	30(8.3,11.8)	23(6.4,8.1)	17(4.7,6.7)	21(5.8,8.2)
DTM		351	26(7.4,9.7)	29(8.3,11.4)	23(6.6,8.1)	13(3.7,5.1)	14(4.0,5.5)
PRO			269	19(7.1,7.5)	21(7.8,7.4)	17(6.3,6.7)	22(8.2,8.6)
OIL				254	11(4.3,3.9)	9(3.5,3.5)	10(3.9,3.9)
IOD					283	133(47.0,52.4)	162(57.2,63.3)
LIO						254	149(58.7,58.2)
LIN							256

The diagonal values show the number of QTL for individual traits. The two values in parenthesis show percentages of pleiotropic QTL of the two traits of the corresponding row and column. YLD, seed yield; DTM, days to maturity; PRO, protein content; OIL, oil content; IOD, iodine value; LIO, linoleic acid content; LIN, linolenic acid content.

**Table 4:** Prediction accuracy (*r* ± *s*) of seven traits using all single nucleotide polymorphisms (SNPs) and different combinations of quantitative trait loci (QTL) identified by different combinations of statistical models. GBLUP was used to estimate *r* values.

Traits	Models	Marker Sets	No. of Markers	*r* ± *s*
YLD	All	QTL of YLD	361	**0.892 ± 0.023a**
	BM	QTL of YLD	323	**0.885 ± 0.027a**
	All	QTL for YLD + DTM	643	**0.879 ± 0.026a**
	BM	QTL of all traits	1208	0.862 ± 0.030b
	All	QTL of all traits	1420	0.860 ± 0.030b
	SS+SM	QTL of all traits	427	0.850 ± 0.031c
	-	All SNPs	17,277	0.841 ± 0.035d
	SS+SM	QTL of YLD	53	0.807 ± 0.034e
	SS	QTL of all traits	133	0.789 ± 0.045f
	SS	QTL of YLD	8	0.483 ± 0.085g
DTM	All	QTL of DTM	351	**0.730 ± 0.062a**
	SS+SM	QTL of DTM	71	**0.720 ± 0.063a**
	BM	QTL of DTM	301	**0.719 ± 0.066a**
	All	QTL for DTM + YLD	643	0.689 ± 0.076b
	BM	QTL of all traits	1208	0.608 ± 0.083b
	All	QTL of all traits	1420	0.603 ± 0.088b
	SS+SM	QTL of all traits	427	0.599 ± 0.087b
	SS	QTL of all traits	133	0.497 ± 0.095c
	-	All SNPs	17,277	0.449 ± 0.101d
	SS	QTL of DTM	28	0.362 ± 0.125e
PRO	All	QTL of PRO	269	**0.894 ± 0.023a**
	BM	QTL of PRO	220	**0.890 ± 0.024a**
	All	QTL for PRO +OIL	504	**0.879 ± 0.026ab**
	SS+SM	QTL of PRO	51	0.877 ± 0.026b
	SS+SM	QTL of all traits	427	0.864 ± 0.031c
	All	QTL of all traits	1420	0.855 ± 0.031d
	BM	QTL of all traits	1208	0.854 ± 0.030d
	-	All SNPs	17,277	0.825 ± 0.034e
	SS	QTL of all traits	133	0.800 ± 0.042f
	SS	QTL of PRO	31	0.681 ± 0.069g
OIL	All	QTL of OIL	254	**0.929 ± 0.016a**
	All	QTL for PRO + OIL	504	**0.927 ± 0.018a**
	SS+SM	QTL of OIL	84	0.919 ± 0.017b
	BM	QTL of OIL	186	0.911 ± 0.023c
	SS+SM	QTL of all traits	427	0.909 ± 0.021c
	All	QTL of all traits	1420	0.909 ± 0.023c
	BM	QTL of all traits	1208	0.907 ± 0.023c
	-	All SNPs	17,277	0.889 ± 0.028d
	SS	QTL of all traits	133	0.845 ± 0.042e
	SS	QTL of OIL	10	0.762 ± 0.058f
IOD	SS+SM	QTL of IOD	72	**0.835 ± 0.041a**
	All	QTL of IOD	283	**0.824 ± 0.046a**
	All	QTL for IOD + LIO + LIN	468	**0.825 ± 0.051a**
	SS+SM	QTL of all traits	427	0.801 ± 0.055b
	BM	QTL of IOD	190	0.752 ± 0.066c
	SS	QTL of IOD	71	0.746 ± 0.065c
	All	QTL of all traits	1420	0.745 ± 0.066c
	BM	QTL of all traits	1208	0.717 ± 0.072d
	SS	QTL of all traits	133	0.717 ± 0.072d
	-	All SNPs	17,277	0.639 ± 0.073e
LIO	All	QTL for IOD + LIO + LIN	468	**0.836 ± 0.043a**
	SS+SM	QTL of LIO	87	**0.835 ± 0.039a**
	All	QTL of LIO	254	**0.834 ± 0.048a**
	SS+SM	QTL of all traits	427	0.817 ± 0.049b
	BM	QTL of LIO	152	0.812 ± 0.049b
	All	QTL of all traits	1420	0.770 ± 0.055c
	SS	QTL of LIO	68	0.765 ± 0.056c
	BM	QTL of all traits	1208	0.744 ± 0.058d
	SS	QTL of all traits	133	0.736 ± 0.066d
	-	All SNPs	17,277	0.672 ± 0.063e
LIN	SS+SM	QTL of LIN	67	**0.837 ± 0.041a**
	All	QTL of LIN	256	**0.833 ± 0.051a**
	All	QTL for IOD + LIO + LIN	468	**0.830 ± 0.047a**
	SS+SM	QTL of all traits	427	0.809 ± 0.053b
	BM	QTL of LIN	170	0.792 ± 0.062c
	SS	QTL of LIN	70	0.756 ± 0.062d
	All	QTL of all traits	1420	0.755 ± 0.061d
	BM	QTL of all traits	1208	0.727 ± 0.066e
	SS	QTL of all traits	133	0.725 ± 0.070e
	-	All SNPs	17,277	0.649 ± 0.069f

Letters indicate significant difference at α= 0.05 level. Tukey’s multiple range test was used. The highest prediction accuracy of each trait is highlighted in bold font. SS, SNP-based single-locus model; SM, SNP-based multi-locus model; BM, block-based model; All, SS+SM+BM; seed yield; DTM, days to maturity; PRO, protein content; OIL, oil content; IOD, iodine value; LIO, linoleic acid content; LIN, linolenic acid content.

**Table 5:** Genomic prediction accuracy (*r* ± *s*) of seven traits affected by different locations.

Trait	Overall			Saskatoon, Saskatchewan			Morden, Manitoba		
	17,277 SNPs	All-Trait QTL	Single-Trait QTL	17,277 SNPs	All-Trait QTL	Single-Trait QTL	17,277 SNPs	All-Trait QTL	Single-Trait QTL
YLD	0.84 ± 0.03 ij	0.86 ±0.03 h	**0.89 ± 0.02 efg**	0.88 ± 0.02 g	0.89 ± 0.02 defg	**0.91 ± 0.02 cde**	0.79 ± 0.04 n	0.82 ± 0.04 lm	**0.85 ± 0.04 hij**
DTM	0.45 ± 0.10 x	0.60 ± 0.09 v	**0.73 ± 0.06 q**	0.51 ± 0.09 w	0.61 ± 0.08 v	**0.70 ± 0.07 r**	0.32 ± 0.12 y	0.52 ± 0.11 w	**0.67 ± 0.07 s**
PRO	0.82 ± 0.03 klm	0.86 ± 0.03 hi	**0.89 ± 0.02 defg**	0.81 ± 0.04 mn	0.84 ± 0.03 ijk	**0.89 ± 0.02 fg**	0.88 ± 0.02 fg	0.90 ± 0.02 cdef	**0.91 ± 0.02 bcd**
OIL	0.89 ± 0.03 fg	0.91 ± 0.02 cd	**0.93 ± 0.02 a**	0.89 ± 0.03 defg	0.91 ± 0.02 bcd	**0.93 ± 0.02 ab**	0.88 ± 0.03 g	0.90 ± 0.02 cdef	**0.92 ± 0.02 abc**
IOD	0.64 ± 0.07 tu	0.75 ± 0.07 p	**0.82 ± 0.05 klm**	0.63 ± 0.07 u	0.74 ± 0.06 pq	**0.82 ± 0.05 lm**	0.66 ± 0.07 st	0.75 ± 0.06 op	**0.83 ± 0.04 jklm**
LIO	0.67 ± 0.06 s	0.77 ± 0.05 o	**0.83 ± 0.05 jkl**	0.67 ± 0.06 s	0.77 ± 0.05 o	**0.83 ± 0.05 jklm**	0.67 ± 0.06 s	0.77 ± 0.05 o	**0.83 ± 0.05 jkl**
LIN	0.65 ± 0.07 tu	0.75 ± 0.06 op	**0.83 ± 0.05 jkl**	0.65 ± 0.07 tu	0.75 ± 0.06 op	**0.82 ± 0.05 klm**	0.65 ± 0.07 stu	0.76 ± 0.06 op	**0.84 ± 0.05 jkl**

The highest prediction accuracy among different marker types is highlighted in bold font. Single-trait QTL, quantitative trait loci (QTL) identified using all models for a specific trait, i.e., a different marker set for each trait; All-trait QTL, all unique QTL identified using all models from all seven traits, i.e., the same marker set for all seven trait; Overall, phenotype BLUEs over four years and two locations, Morden, Manitoba and Saskatoon, Saskatchewan; YLD, seed yield; DTM, days to maturity; PRO, protein content; OIL, oil content; IOD, iodine value; LIO, linoleic acid content; LIN, linolenic acid content; SNP, single nucleotide polymorphism. The letters after *r* ± s values represent statistical significance of *r* values among 63 combinations of seven traits, three marker sets, and three location levels (two locations plus overall BLUEs over two locations).

**Table 6:** Broad-sense and genomic heritability of seven traits.

Trait	Broad-Sense Heritability (*H*^2^)	Genomic Heritability Based on Single Trait QTL (*h*^2^)	Genomic Heritability Based on 1420 QTL of 7 Traits (*h*^2^)	Genomic Heritability Based on 17,277 SNPs (*h*^2^)	Maximum Perdition Accuracy (*r*)	Relative Efficiency (r/*H*^2^)
YLD	0.20 ± 0.02	0.68 ± 0.06	0.62 ± 0.08	0.62 ± 0.09	0.89 ± 0.02	4.45
DTM	0.49 ± 0.03	0.58 ± 0.08	0.59 ± 0.09	0.46 ± 0.11	0.73 ± 0.06	1.49
PRO	0.44 ± 0.04	0.71 ± 0.08	0.62 ± 0.08	0.62 ± 0.09	0.89 ± 0.02	2.02
OIL	0.69 ± 0.03	0.66 ± 0.06	0.72 ± 0.07	0.73 ± 0.07	0.93 ± 0.02	1.35
IOD	0.81 ± 0.02	0.73 ± 0.05	0.73 ± 0.07	0.72 ± 0.07	0.84 ± 0.04	1.04
LIO	0.84 ± 0.02	0.73 ± 0.05	0.74 ± 0.07	0.74 ± 0.07	0.84 ± 0.04	1.00
LIN	0.83 ± 0.02	0.76 ± 0.05	0.73 ± 0.07	0.73 ± 0.07	0.84 ± 0.04	1.01

YLD, seed yield; DTM, days to maturity; PRO, protein content; OIL, oil content; IOD, iodine value; LIO, linoleic acid content; LIN, linolenic acid content; SNP, single nucleotide polymorphism; QTL, quantitative trait loci.

## Supplementary Materials

Supplementary materials can be found at https://www.mdpi.com/1422-0067/21/5/1577/s1. Table S1. Results of analysis of variance (ANOVA) for the seven traits; Table S2. Quantitative trait nucleotides (QTNs)/quantitative trait loci (QTL) identified for the seven traits; Table S3. Average allele effects of quantitative trait loci (QTL) identified by different statistical models for all the seven traits; Table S4. Summary statistics of quantitative trait loci (QTL) identified by seven multi-locus models for the seven traits; Table S5. Analysis of variance for genomic prediction accuracy (*r*) of genomic selection models constructed by different traits, statistical models and marker sets; Table S6. Analysis of variance (ANOVA) for genomic prediction accuracy (*r*) in terms of locations (Morden and Saskatoon, Canada, average of two locations), traits (YLD, DTM, PRO, OIL, IOD, LIO and LIN), and marker types (all SNPs, QTL of single traits and QTL of all traits); Table S7. Pearson correlation coefficients of phenotypes among the seven traits.

## Abbreviations

DTM	days to maturity
GBS	genotype by sequencing
GEBV	genomic estimate of breeding value
GWAS	genome-wide association study
IOD	iodine value
LD	linkage disequilibrium
LIN	linolenic acid
LIO	linoleic acid
MAF	minor allele frequency
OIL	oil content
QTN	quantitative trait nucleotide
QTL	quantitative trait locus/loci
SNP	single nucleotide polymorphism
YLD	seed yield
