# Supplementary material for: Genomic Prediction Accuracy of Seven Breeding Selection Traits Improved by QTL Identification in Flax
Source: Int J Mol Sci. 2020 Feb 25;21(5):1577. doi: 10.3390/ijms21051577 (PMC7084455; doi:10.3390/ijms21051577)
Supplement: Supplementary file 1 [file ijms-21-01577-s001.zip › ijms-717933 suppl for final/Table_S1,3,4,5,6,7.pdf]

Table S1. Results of analysis of variance (ANOVA) for seven traits.

Trait	Source	DF	Sum of Square (SS)	Mean SS	F	Pr(> F)
YLD	Location	2	137.0	68.5	583.1	<2e-16
	Year	4	255.7	63.9	544.2	<2e-16
	Location * Year	8	229.3	28.7	244.0	<2e-16
	Residuals	3386	397.7	0.1		
DTM	Location	2	19,772.0	9886.0	878.3	<2e-16
	Year	3	49,889.0	16,630.0	1477.5	<2e-16
	Location * Year	6	1647.0	274.0	24.4	<2e-16
	Residuals	2941	33,102.0	11.0		
PRO	Location	2	8,332.0	4166.0	1688.2	<2e-16
	Year	3	3,055.0	1018.0	412.6	<2e-16
	Location * Year	6	2,585.0	431.0	174.6	<2e-16
	Residuals	2615	6,453.0	2.0		
OIL	Location	2	3558.0	1,779.1	306.7	<2e-16
	Year	4	2518.0	629.4	108.5	<2e-16
	Location * Year	8	343.0	42.9	7.4	8.45E-10
	Residuals	3388	19,655.0	5.8		
IOD	Location	2	68,719.0	34,359.0	173.7	<2e-16
	Year	4	33,202.0	8300.0	42.0	<2e-16
	Location * Year	8	15,339.0	1917.0	9.7	2.20E-13
	Residuals	3388	670,097.0	198.0		
LIO	Location	2	1718.0	859.0	3.3	0.0383 (NS)
	Year	4	31,571.0	7893.0	30.0	<2e-16
	Location * Year	8	55.0	7.0	0.02	1 (NS)
	Residuals	3388	891,244.0	263.0		
LIN	Location	2	14,120.0	7060.0	28.7	4.40E-13
	Year	4	31,867.0	7967.0	32.4	<2e-16
	Location * Year	8	4,891.0	611.0	2.5	0.011
	Residuals	3388	833,644.0	246.0		

Table S3. Average allele effects of QTL identified by different statistical models for all the seven traits.

Model type	Model	No. of QTL	Average R^2 of QTL
SS	GLM	133	3.74 ± 6.12
	MLM	31	3.46 ± 4.52
SM	FarmCPU	96	4.71 ± 7.58
	FASTmrEMMA	52	5.22 ± 8.03
	FASTmrMLM	96	4.84 ± 7.69
	ISIS EM-BLASSO	113	6.09 ± 8.02
	mrMLM	121	5.32 ± 8.65
	pKWmEB	133	5.26 ± 6.51
	pLARmEB	130	4.78 ± 7.64
BM	RTM-GWAS	1,208	3.37 ± 5.35

SS, single SNP based single locus model; SM, single SNP based multi-locus model; BM, haplotype block based multi-locus model;.

Table S4. Summary statistics of QTL identified by seven multi-locus models for the seven traits.

Category	Model	YLD	DTM	PRO	OIL	LOD	LIO	LIN	All traits
Total	FarmCPU (total)	6	19	15	11	23	35	19	96
	FASTmrEMMA (total)	12	9	11	13	7	8	8	52
	FASTmrMLM (total)	17	19	11	29	18	18	16	96
	ISIS EM-BLASSO (total)	16	28	21	28	19	13	12	113

	mrMLM (total)	26	27	19	47	25	28	27	151
	pKWmEB (total)	21	25	17	28	26	21	19	133
	pLARmEB (total)	13	19	20	33	28	31	29	130
	Total	**53**	**71**	**51**	**84**	**72**	**87**	**67**	**355**
Unique to single model	FarmCPU	3	11	6	4	11	23	6	47
	FASTmrEMMA	3	2	1	2	1	1	2	6
	FASTmrMLM	3	3	0	3	3	1	3	10
	ISIS EM-BLASSO	8	10	6	7	8	6	4	33
	mrMLM	3	7	2	7	4	9	7	25
	pKWmEB	4	8	8	12	11	10	9	44
	pLARmEB	3	2	6	5	5	13	11	29
	Sub total	**27**	**43**	**29**	**40**	**43**	**63**	**42**	**194**
Shared by two models	FarmCPU,ISIS EM-BLASSO	0	0	1	0	0	0	0	2
	FarmCPU,mrMLM	0	0	0	0	0	0	1	1
	FarmCPU,pKWmEB	0	1	0	1	0	0	0	3
	FarmCPU,pLARmEB	0	1	0	0	2	1	4	5
	FASTmrEMMA,FASTmrMLM	0	0	0	1	0	0	0	1
	FASTmrEMMA,pKWmEB	1	0	1	2	0	1	1	4
	FASTmrEMMA,pLARmEB	1	0	0	0	0	0	0	0
	FASTmrMLM,FarmCPU	0	0	0	0	1	0	0	0
	FASTmrMLM,ISIS EM-BLASSO	0	0	0	1	0	0	0	2
	FASTmrMLM,mrMLM	4	3	0	2	1	1	0	7
	FASTmrMLM,pKWmEB	2	0	0	0	0	0	1	1
	FASTmrMLM,pLARmEB	0	0	0	1	0	1	2	1
	ISIS EM-BLASSO,mrMLM	0	0	0	0	0	0	1	0
	ISIS EM-BLASSO,pKWmEB	1	1	0	2	3	2	3	9
	ISIS EM-BLASSO,pLARmEB	0	2	0	1	1	1	0	4
	mrMLM,pKWmEB	0	0	0	0	0	0	0	4
	mrMLM,pLARmEB	0	0	1	5	2	1	0	6
	pKWmEB,pLARmEB	2	2	0	1	2	0	0	5
	Sub total	**11**	**10**	**3**	**17**	**12**	**8**	**13**	**55**
Shared by three models	FarmCPU,ISIS EM-BLASSO,mrMLM	1	0	0	1	0	0	0	0
	FarmCPU,ISIS EM-BLASSO,pKWmEB	0	1	0	0	0	0	0	1
	FarmCPU,ISIS EM-BLASSO,pLARmEB	0	1	1	0	0	0	0	2
	FarmCPU,mrMLM,pLARmEB	0	0	1	0	0	0	0	2
	FarmCPU,pKWmEB,pLARmEB	0	0	0	0	1	0	0	1
	FASTmrEMMA,FarmCPU,pLARmEB	0	1	0	0	0	0	0	1
	FASTmrEMMA,FASTmrMLM,ISIS EM-BLASSO	0	0	0	1	0	0	0	1
	FASTmrEMMA,FASTmrMLM,mrMLM	2	0	0	1	0	0	0	3
	FASTmrEMMA,FASTmrMLM,pLARmEB	0	0	0	1	0	1	1	1
	FASTmrEMMA,ISIS EM-BLASSO,mrMLM	0	0	1	0	0	0	0	1
	FASTmrEMMA,ISIS EM-BLASSO,pKWmEB	0	0	1	0	0	0	0	2
	FASTmrEMMA,mrMLM,pKWmEB	1	0	0	0	0	0	0	1
	FASTmrEMMA,pKWmEB,pLARmEB	1	0	0	0	1	0	0	2
	FASTmrMLM,FarmCPU,mrMLM	0	1	0	1	0	1	0	2
	FASTmrMLM,FarmCPU,pKWmEB	0	0	0	0	0	0	0	2
	FASTmrMLM,FarmCPU,pLARmEB	0	0	0	1	0	0	1	1
	FASTmrMLM,ISIS EM-BLASSO,mrMLM	0	1	1	1	0	0	0	1
	FASTmrMLM,ISIS EM-BLASSO,pLARmEB	0	0	0	0	1	1	0	2
	FASTmrMLM,mrMLM,pKWmEB	2	1	0	1	0	0	0	4
	FASTmrMLM,mrMLM,pLARmEB	0	0	1	2	2	1	1	3
	FASTmrMLM,pKWmEB,pLARmEB	0	0	0	0	0	1	0	1
	ISIS EM-BLASSO,mrMLM,pKWmEB	0	0	1	0	0	0	0	1
	ISIS EM-BLASSO,mrMLM,pLARmEB	0	0	1	3	0	0	0	5
	ISIS EM-BLASSO,pKWmEB,pLARmEB	0	0	0	0	1	0	0	1
	mrMLM,pKWmEB,pLARmEB	2	0	0	1	1	0	1	4
	Sub total	**9**	**6**	**8**	**14**	**7**	**5**	**4**	**45**

Shared by four models	FarmCPU,ISIS EM-BLASSO,mrMLM,pKWmEB	0	0	1	1	0	0	0	2
	FarmCPU,ISIS EM-BLASSO,pKWmEB,pLARmEB	0	0	1	0	0	0	0	1
	FarmCPU,mrMLM,pKWmEB,pLARmEB	0	0	0	0	0	0	1	0
	FASTmrEMMA,FarmCPU,pKWmEB,pLARmEB	0	0	0	0	0	1	0	1
	FASTmrEMMA,FASTmrMLM,FarmCPU,pLARmEB	0	0	0	0	1	0	0	1
	FASTmrEMMA,FASTmrMLM,ISIS EM-BLASSO,pKWmEB	0	1	0	0	0	0	0	1
	FASTmrEMMA,FASTmrMLM,ISIS EM-BLASSO,pLARmEB	0	0	1	1	0	0	0	2
	FASTmrEMMA,FASTmrMLM,mrMLM,pKWmEB	0	0	1	0	0	0	0	2
	FASTmrEMMA,FASTmrMLM,mrMLM,pLARmEB	0	0	0	1	0	1	0	2
	FASTmrEMMA,ISIS EM-BLASSO,pKWmEB,pLARmEB	0	1	0	0	0	0	0	1
	FASTmrMLM,FarmCPU,ISIS EM-BLASSO,pKWmEB	1	0	0	0	0	0	0	0
	FASTmrMLM,FarmCPU,mrMLM,pLARmEB	0	0	1	0	2	2	1	3
	FASTmrMLM,FarmCPU,pKWmEB,pLARmEB	0	0	0	0	0	1	0	0
	FASTmrMLM,ISIS EM-BLASSO,mrMLM,pKWmEB	1	1	0	0	1	0	0	2
	FASTmrMLM,ISIS EM-BLASSO,mrMLM,pLARmEB	1	1	0	2	1	0	0	5
	FASTmrMLM,ISIS EM-BLASSO,pKWmEB,pLARmEB	0	0	0	1	0	0	0	1
	FASTmrMLM,mrMLM,pKWmEB,pLARmEB	0	0	0	1	0	0	0	2
	Sub total	**3**	**4**	**5**	**7**	**5**	**5**	**2**	**26**
Shared by five models	ISIS EM-BLASSO,mrMLM,pKWmEB,pLARmEB	0	1	0	0	0	0	0	1
	FASTmrEMMA,FASTmrMLM,FarmCPU,ISIS EM-BLASSO,pKWmEB	0	0	0	0	0	1	0	0
	FASTmrEMMA,FASTmrMLM,FarmCPU,mrMLM, pLARmEB	0	0	0	0	0	1	0	1
	FASTmrEMMA,FASTmrMLM,ISIS EM-BLASSO,mrMLM,pLARmEB	0	0	1	1	0	0	1	1
	FASTmrEMMA,FASTmrMLM,mrMLM,pKWmEB, pLARmEB	0	0	1	0	0	0	0	0
	FASTmrEMMA,ISIS EM-BLASSO,mrMLM,pKWmEB,pLARmEB	1	0	0	0	0	0	0	1
	FASTmrMLM,FarmCPU,ISIS EM-BLASSO,mrMLM,pLARmEB	0	0	1	0	0	0	1	2
	FASTmrMLM,FarmCPU,ISIS EM-BLASSO,pKWmEB,pLARmEB	0	0	0	1	0	0	0	1
	FASTmrMLM,FarmCPU,mrMLM,pKWmEB,pLARmEB	0	0	0	0	0	1	1	2
	FASTmrMLM,ISIS EM-BLASSO,mrMLM,pKWmEB,pLARmEB	0	3	0	1	0	0	0	7
	Sub total	**1**	**4**	**3**	**3**	**0**	**3**	**3**	**16**
Shared by six models	FASTmrEMMA,FarmCPU,ISIS EM-BLASSO,mrMLM,pKWmEB,pLARmEB	1	0	0	0	0	0	0	1
	FASTmrEMMA,FASTmrMLM,FarmCPU,ISIS EM-BLASSO,mrMLM,pLARmEB	0	0	1	0	0	0	1	1
	FASTmrEMMA,FASTmrMLM,FarmCPU,ISIS EM-BLASSO,pKWmEB,pLARmEB	0	0	0	0	1	0	1	1
	FASTmrEMMA,FASTmrMLM,FarmCPU,mrMLM, pKWmEB,pLARmEB	0	0	0	0	2	1	1	1
	FASTmrEMMA,FASTmrMLM,ISIS EM-BLASSO,mrMLM,pKWmEB,pLARmEB	1	2	1	2	0	0	0	7
	FASTmrMLM,FarmCPU,ISIS EM-BLASSO,mrMLM,pKWmEB,pLARmEB	0	0	0	1	1	2	0	3
	Sub total	**2**	**2**	**2**	**3**	**4**	**3**	**3**	**14**

Shared by seven models	FASTmrEMMA,FASTmrMLM,FarmCPU,ISIS EM-BLASSO,mrMLM,pKWmEB,pLARmEB	**0**	**2**	**1**	**0**	**1**	**0**	**0**	**5**

Table S5. Analysis of variance (ANOVA) for genomic prediction accuracy (r) of genomic selection models constructed by different traits, statistical models of GWAS and marker sets.

Source	DF	Sum of Square	Mean Square	F Value	P (> F)
GWAS Models	4	95.32	23.83	7497.8	<2e-16
Traits	6	239.32	39.89	12550.3	<2e-16
Marker types	1	1.46	1.46	460.7	<2e-16
GWAS models × Traits	24	51.37	2.14	673.5	<2e-16
GWAS models × Marker types	3	21.73	7.24	2279.2	<2e-16
Traits × Marker sets	6	13.84	2.31	725.9	<2e-16
GWAS models × Traits × Marker types	18	16.58	0.92	289.8	<2e-16
Error	31,437	99.91			

Table S6. Analysis of variance (ANOVA) for genomic prediction accuracy (*r*) in terms of locations (Morden and Saskatoon, Canada, average of two locations), traits (YLD, DTM, PRO, OIL, IOD, LIO and LIN), and marker types (all SNPs, QTL of single traits and QTL of all traits.

Source	*DF*	*F*	*P*
Location (L)	2	129.86	$<2.2^{-16}$
Traits (T)	6	12939.93	$<2.2^{-16}$
Marker types (M)	2	10927.98	$<2.2^{-16}$
L × T	12	214.87	$<2.2^{-16}$
L × M	4	23.93	$<2.2^{-16}$
T x M	12	695.59	$<2.2^{-16}$
L × T × M	12	24.2	$<2.2^{-16}$
Error	20937		
Total	20987		

Table S7. Pearson correlation coefficients of phenotypes among the seven traits.

Trait	DTM	PRO	OIL	IOD	LIO	LIN
YLD	0.27**	−0.73 **	0.57 **	0.46 **	−0.51 **	0.49 **
DTM		−0.45 **	0.24 **	0.13	−0.22 **	0.18 *
PRO			−0.70 **	−0.52 **	0.52 **	−0.52 **
OIL				0.37 **	−0.27 **	0.31 **
IOD					−0.95 **	0.99 **
LIO						−0.99 **

* and ** represent statistical significance at 0.05 and 0.01 probability level, respectively. seed yield; DTM, days to maturity; PRO, protein content; OIL, oil content; IOD, iodine value; LIO, linoleic acid content; LIN, linolenic acid content.
